# Targeted Collection of Plasmid DNA in Large and Growing Animal Muscles 6 Weeks after DNA Vaccination with and without Electroporation

**DOI:** 10.1155/2015/326825

**Published:** 2015-08-25

**Authors:** Daniel Dory, Vincent Le Moigne, Roland Cariolet, Véronique Béven, André Keranflec'h, André Jestin

**Affiliations:** ^1^French Agency for Food, Environmental and Occupational Health Safety (ANSES), Viral Genetics and Biosafety Unit, 22440 Ploufragan, France; ^2^French Agency for Food, Environmental and Occupational Health Safety (ANSES), Pathogen-Free Pig Breeding and Testing Facility, 22440 Ploufragan, France

## Abstract

DNA vaccination has been developed in the last two decades in human and animal species as a promising alternative to conventional vaccination. It consists in the injection, in the muscle, for example, of plasmid DNA encoding the vaccinating polypeptide. Electroporation which forces the entrance of the plasmid DNA in cells at the injection point has been described as a powerful and promising strategy to enhance DNA vaccine efficacy. Due to the fact that the vaccine is composed of DNA, close attention on the fate of the plasmid DNA upon vaccination has to be taken into account, especially at the injection point. To perform such studies, the muscle injection point has to be precisely recovered and collected several weeks after injection. This is even more difficult for large and growing animals. A technique has been developed to localize precisely and collect efficiently the muscle injection points in growing piglets 6 weeks after DNA vaccination accompanied or not by electroporation. Electroporation did not significantly increase the level of remaining plasmids compared to nonelectroporated piglets, and, in all the cases, the levels were below the limit recommended by the FDA to research integration events of plasmid DNA into the host DNA.

## 1. Introduction

DNA vaccination is widely studied to develop new and alternative vaccines for humans and animals. DNA vaccines are circular plasmid DNA molecules that encode the vaccinating antigens, these antigens being synthesized inside cells of the injected body. Many efforts have been made to increase the immunising potential of these vaccines. For example, plasmids encoding cytokines or copresentation molecules as well as toll-like receptors agonists were successfully used as adjuvants in various models (for a review see [1]). Other strategies were based on the route of injection, the controlled release of the plasmids, and/or the forcing of the entrance of the plasmids in the cells at the injection point. Among the strategies that force the entrance of the plasmids, electroporation has a promising future [2]. Electroporation consists in the application of an electric current on both sides of the injection point. Cells at the injection site are thereby temporarily permeabilized, promoting the entry of plasmids conveyed by the electric current into the cells. This results in many cases in the improvement of DNA vaccine efficacies [3–6]. In particular, electroporation has been demonstrated as a powerful technique also in large animals, including pigs [7–9].

DNA vaccination is generally well tolerated, even when electroporation is applied [9]. No adverse reactions and changes in metabolic activity were observed in numerous animal and human clinical trials upon DNA vaccination [10]. Histological damage has been hardly observed, with the exception, for example, of one study in rats shortly after the injection, but this was associated with the route of injection [11].

Due to the fact that DNA vaccines are composed of DNA, close attention on the fate of the plasmid DNA upon vaccination has to be taken into account. In general, most plasmids remain at the site of injection (for a review see [12]) for, in certain cases, up to several months [13]. Shortly after injection, small amounts of plasmids spread throughout the body and are detected in remote organs [14]. DNA vaccination involves the introduction of small amounts of plasmid DNA into the nucleus of host cells. It is then conceivable that there is a potential risk of partial or complete integration of plasmid DNA into the host cell genome. Therefore, this potential risk should be examined carefully. Furthermore, it is also conceivable that for techniques allowing an entrance increase of the plasmids in the cells, as it is the case for electroporation, these potential risks should be even more deeply taken into consideration. Usually, plasmids are quantified in the injected tissue samples by PCR-based methods (as, e.g., in [15]). It is admitted that if there are no plasmids detected, plasmid integration event may not have occurred. To unambiguously characterize integration events, Wang et al. developed in mice the PCR-based repeat anchored integration capture-PCR (RAIC-PCR) [16]. Four integration events have been identified four weeks after intramuscular injection of the DNA vaccine followed by electroporation.

When small animal models like mice are used, the fate of DNA plasmids can be studied on whole injected muscle homogenates. To apply these PCR-based tests for large and growing animal models (e.g., pigs), it is essential to recover the injection point, especially several weeks after injection. In fact, the muscle and skin surfaces of the animals are large and growing. Therefore, precise benchmarks are essential to identify the injection points. In the present study, we developed a strategy to be able to localize precisely the injection point in muscles of growing piglets at least 6 weeks after DNA vaccination. With our strategy, the benchmarks are not located directly within the injection point; therefore there is no disturbance of the injection point due to the presence of these benchmarks. With this technique, we compared the concentration of remaining plasmids 6 weeks after a single DNA vaccination of piglets accompanied or not by electroporation. The model used here is a DNA vaccine against pseudorabies virus infection. The electroporation conditions used here were previously shown to be efficient to induce a significant increase of immune responses due to the DNA vaccine.

## 2. Materials and Methods

### 2.1. Plasmids

The endotoxin free pcDNA3 plasmids encoding or not pseudorabies virus glycoprotein B (PrV-gB-pcDNA3) were produced and purified as previously described [9, 17].

### 2.2. Pig Experiments

**Figure 1:**
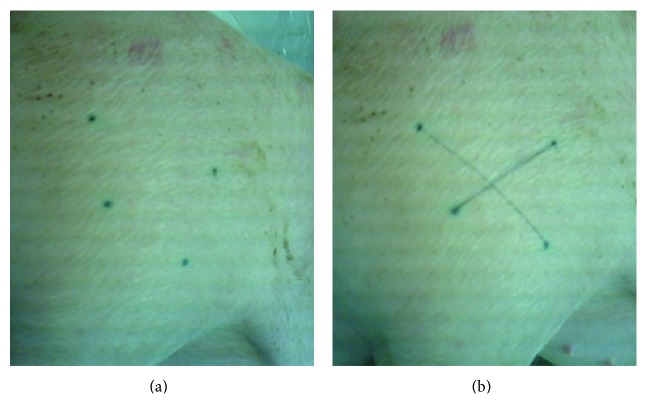
Identification of the injection point. (a) Four dots were tattooed with Indian ink on the skin of the left* biceps femoris* muscle 2 to 3 weeks before the injection. (b) The injection site of the plasmids was located at the intersection of the two lines passing through these dots. These two lines were drawn on the skin just before the injection.

**Figure 2:**
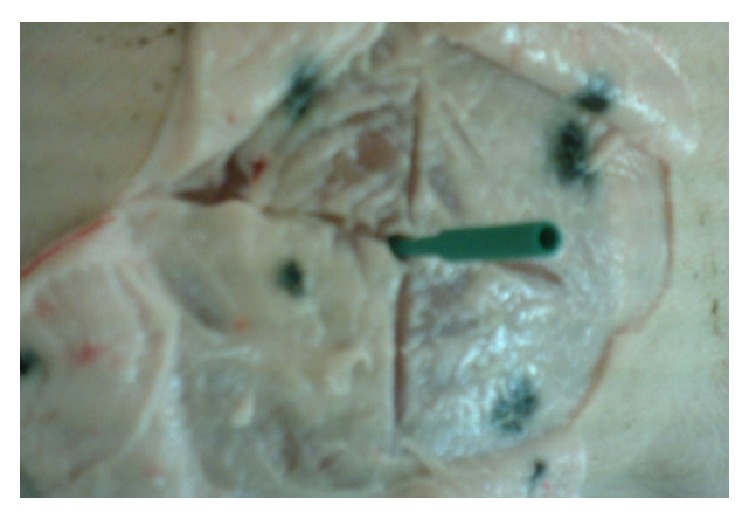
Sampling of the injection point 6 weeks after injection. Six weeks after injection, the injection point was localized as done at the injection time (Figure 1). The two lines were drawn thanks to the four tattooed points. After removing the skin and the fat layer, the portion of the injected muscle was sampled using a disposable 2 cm long biopsy punch that was horizontally applied on the muscle surface.

The experimental protocol was approved by the ethic committee for animal experimentation of ANSES/National Veterinary School of Alfort/University of Paris-Est Créteil (France) (Notice number 10/04/13-05). Pigs were housed and treated in accordance with the requirements of the local veterinary authority. Four groups of four specific pathogen-free eight-week old pigs were used. The injection site of the plasmids was identified through four dots tattooed with Indian ink on the skin of the left* biceps femoris* muscle, the injection site being located at the intersection of the two lines passing through these dots (Figure 1). All pigs were anesthetized with an auricular intravenous injection of thiopental (1 g/50 kg body weight). The first and second group were injected with 2.5 × 10^14^ copies of PrV-gB-pcDNA3 prepared in 600 *μ*L PBS. The third one received 2.5 × 10^14^ copies of pcDNA3 and the last group was injected with PBS. 0.45 mm × 12 mm needles were used. Eighty seconds later [18], electroporation which consists of 5 pulses of 150 V and 20 ms with a 200 ms interval between each pulse [7] was applied through stainless-steel electrodes (0.2 mm wires, 1 cm long, and 10 mm apart) introduced on either side of the injection point of pigs of groups 2 to 4. The electric current was applied with a BTX ECM 830 pulse generator (Harvard Apparatus, Holliston, MA, USA). Pigs were observed daily. Body temperature and body weight were measured daily and weekly, respectively. Pigs were sacrificed six weeks after injection. The muscle injection site identified through the tattooed dots was sampled using a disposable 2 cm long and 0.8 cm diameter biopsy punch (Figure 2), frozen in liquid nitrogen, and stored at −80°C until DNA extraction.

### 2.3. DNA Extraction and Quantification of Plasmids by Quantitative PCR (qPCR)

Prior to DNA extraction, the 2 cm long pieces of muscle excised were divided into six equal samples (from the superior to the inferior part of the muscle). Each muscle fraction was weighed and resuspended in PBS buffer according to the measured mass. Then homogenization was carried out using a Teflon pestle at 30 Hz for 1 min or until all major tissue clumps were dispersed. Host DNA extractions were performed on 30 mg of the homogenized tissue sample using the QIAamp DNA Mini Kit (Qiagen) after overnight proteinase *K* digestion according to the manufacturer's instructions. Thereafter, plasmid DNA concentration was measured in each muscle sample by quantitative PCR (qPCR). The target of the qPCR is a 92 nucleotides sequence located in the neomycin gene of the plasmids. Primers, probes, and qPCR conditions were those previously described [19]. Measurements were performed in triplicate. Experimental data were analyzed using the nonparametric Mann-Whitney test [20] included in SYSTAT 9 software (SYSTAT Software, Inc., Point Richmond, CA, USA).

## 3. Results and Discussion

**Figure 3:**
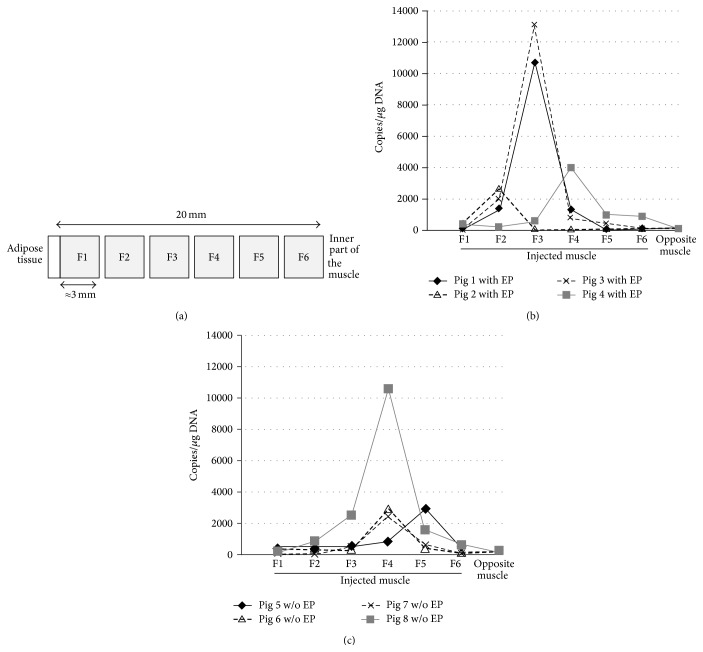
Plasmid concentrations in the different muscle fractions six weeks after injection. Two groups of four pigs were intramuscularly injected with 2.5 × 10^14^ copies of PrV-gB-pcDNA3 with or without electroporation, respectively. Six weeks later, excision of the muscle injection site was performed using a disposable 2 cm long and 0.8 cm diameter biopsy punch. (a) The excised muscle portion was divided into six fractions, F1 to F6. Fraction F1 represents the most external part of the muscle (i.e., under the skin) and F6 the most internal part. Thereafter plasmid DNA concentration was measured in each fraction after DNA extraction. Levels (in number of plasmid copies per *μ*g of total DNA) of PrV-gB-pcDNA3 present in injected or opposite (noninjected)* biceps femoris* were quantified by real-time qPCR. Individual plasmid concentrations in each muscle sample for each pig injected with (b) or without (c) electroporation are presented. When taking into account the mean values for each fraction (not shown), the differences between both groups were not statistically significant (*P* > 0.05, nonparametric Mann-Whitney test).

Four points were tattooed with Indian ink on the skin of the left* biceps femoris* muscle of 5-week-old piglets (as shown in Figure 1). When the piglets were eight-week old, they were i.m. injected either with PrV-gB-pcDNA3 followed or not by electroporation, with pcDNA3 and electroporation, or with PBS and electroporation. The injection site was located at the intersection of the two lines passing through these dots (Figure 1). The mean weight of the animals at the vaccination time was of 24.7 ± 2.1 kg. No adverse reactions, no fever, and no delay in the growth of the pigs were observed during the 6-week period following these injections. The mean weight of the animals 6 weeks after injection was 66.5 ± 5.4 kg. This means that the piglets gained 41.8 ± 4.0 kg during the whole experimental period (i.e., about 170% of their initial weight). In a previous report we showed that plasmid injection coupled with electroporation applied exactly in the same manner as here increased the production of specific antibodies against PrV and peripheral blood mononuclear cells proliferated in response to stimulation with PrV glycoproteins [9]. This means that the electroporation conditions used here are effective ones. At week 6 after injection, 2 cm long muscle samples were collected exactly at the injection site using the dots tattooed on the skin (Figure 1). This time-point was the same as used in the study describing the research of integration events by RAIC-PCR [16]. Furthermore, this time-point seemed to us realistic since we detected small amounts of plasmids in the injected muscle 21 days after DNA vaccination, without electroporation, with much less plasmids injected, and without identifying precisely the injection point as done here [14]. At this time-point, the four tattooed dots were still strongly marked. The two lines delineating the injection site were drawn again on the skin (as shown in Figure 1). After removing the skin and the fat layer, the portion of the injected muscle was sampled using a disposable 2 cm long and 0.8 cm diameter biopsy punch that was horizontally applied on the muscle surface (Figure 2), frozen in liquid nitrogen, and stored at −80°C until DNA extraction. In preliminary experiments, the injection point was found to be located approximately in the middle of the muscle sample (data not shown). No plasmids were detected in pigs injected with PBS. The fractions containing the highest concentration of plasmids (around 3,000 to 14,000 copies/*μ*g of host DNA) are located around the middle of the muscle samples, between fractions F2 and F5, with 6 out of 8 pigs within fractions F3 or F4 (Figure 3). The concentrations were higher in F2–F4 (1 pig in F2, 2 pigs in F3, and 1 pig in F4) when electroporation was applied and in F4-F5 (3 pigs in F4 and 1 pig in F5), that is, deeper, in the other case. When taking into account the mean values for each fraction, the concentrations of plasmids within the electropored muscle fractions were not significantly higher than in the nonelectropored ones (*P* > 0.05) (not shown). But these observations have to be taken with caution since the pressure we applied to the device was not controlled, although we tried. Nevertheless this seems consistent with the fact that less diffusion of the plasmids and better precision of injection are observed with electroporation [21]. Importantly, the fraction which is at the end of the needle is restricted to a small area, at least in depth, which shows the usefulness of precise benchmarks. Finally all the electropored and nonelectropored muscle samples have a concentration of plasmid DNA inferior to 30,000 copies/*μ*g of host DNA. If we take into account the recommendations of the FDA [22], it is not necessary to perform additional integration analyses of plasmid DNA into host DNA since the probability of integration is low (<30,000 copies of plasmid/*μ*g of host DNA).

In conclusion, a method to recover at least in depth the DNA vaccine injection area 6 weeks after injection in growing piglets was developed. At the time of vaccination, the mean weight of the piglets was 24.7 ± 2.1 kg and at the sampling time it was 66.5 ± 5.4 kg. This means that between these two time-points the piglets gained around 40 kg (+140%). Even if electroporation enables significant increases of immune responses levels [9], no significant enhancement of remaining plasmids was observed when electroporation was applied compared to nonelectropored piglets. Furthermore, as electroporation consists in the forcing of the plasmid entrance in cells, special attention has to be paid on the potential risk of integration of plasmid DNA in host DNA. This method described here will be useful to obtain porcine muscle fractions to further study the fate of the plasmids upon DNA vaccination in evaluating their integration within host DNA if the level of remaining plasmids is above 30.000 copies/*μ*g of host DNA (according to the FDA recommendations) [22].
